# Interleukin-32 Promotes Osteoclast Differentiation but Not Osteoclast Activation

**DOI:** 10.1371/journal.pone.0004173

**Published:** 2009-01-12

**Authors:** Guillaume Mabilleau, Afsie Sabokbar

**Affiliations:** Nuffield Department of Orthopaedic Surgery, University of Oxford, Oxford, United Kingdom; Yale University School of Medicine, United States of America

## Abstract

**Background:**

Interleukin-32 (IL-32) is a newly described cytokine produced after stimulation by IL-2 or IL-18 and IFN-γ. IL-32 has the typical properties of a pro-inflammatory mediator and although its role in rheumatoid arthritis has been recently reported its effect on the osteoclastogenesis process remains unclear.

**Methodology/Principal Findings:**

In the present study, we have shown that IL-32 was a potent modulator of osteoclastogenesis *in vitro*, whereby it promoted the differentiation of osteoclast precursors into TRAcP+ VNR+ multinucleated cells expressing specific osteoclast markers (up-regulation of NFATc1, OSCAR, Cathepsin K), but it was incapable of inducing the maturation of these multinucleated cells into bone-resorbing cells. The lack of bone resorption in IL-32-treated cultures could in part be explain by the lack of F-actin ring formation by the multinucleated cells generated. Moreover, when IL-32 was added to PBMC cultures maintained with soluble RANKL, although the number of newly generated osteoclast was increased, a significant decrease of the percentage of lacunar resorption was evident suggesting a possible inhibitory effect of this cytokine on osteoclast activation. To determine the mechanism by which IL-32 induces such response, we sought to determine the intracellular pathways activated and the release of soluble mediators in response to IL-32. Our results indicated that compared to RANKL, IL-32 induced a massive activation of ERK1/2 and Akt. Moreover, IL-32 was also capable of stimulating the release of IL-4 and IFN-γ, two known inhibitors of osteoclast formation and activation.

**Conclusions/Significance:**

This is the first *in vitro* report on the complex role of IL-32 on osteoclast precursors. Further clarification on the exact role of IL-32 *in vivo* is required prior to the development of any potential therapeutic approach.

## Introduction

Interleukin-32 (IL-32) is a newly described cytokine produced mainly by T-, natural killer, epithelial cells and monocytes after stimulation by Interleukin-2, Interleukin-18 or IFN-γ [1], [2]. It was formerly known as natural killer cell transcript 4 [3]. Although IL-32 can bind proteinase 3, a neutrophil-derived serine protease, its receptor is unknown [4], [5]. IL-32 has the typical properties of a pro-inflammatory mediator by stimulating TNF-α, IL-1β and IL-8 production and by activating the NF-κB and p38 mitogen-activated protein (MAP) kinase pathways [1]. IL-32 seems to be involved in a variety of diseases such as inflammatory bowel disease, myelodysplastic syndrome and chronic myelomonocytic leukaemia, HIV infection [6]–[8]. Moreover, it has been reported recently that in a cohort of patient suffering with rheumatoid arthritis, IL-32 was significantly increased in the synovial tissue and that its levels were strongly correlated with the severity of the disease [9], [10]. Moreover, IL-32 has been demonstrated to induce joint inflammation with concomitant mild cartilage damage when injected intra-articularly in murine knee joints [9]. Despite these observations, the impact of this cytokine on osteoclastogenesis remains unclear.

Osteoclasts are multinucleated cells originating from the hematopoietic lineage (CFU-GM) that specifically function in lacunar bone resorption [11], [12]. Osteoclast differentiation from circulating hematopoietic precursors requires the presence of macrophage colony-stimulating factor (M-CSF) and the receptor activator for nuclear factor κB ligand – RANKL [13]–[16]. RANKL is a member of the tumor necrosis factor (TNF) superfamily that is expressed on osteoblasts and T-cells and interacts with its receptor, RANK, expressed on osteoclast precursors [15], [17]–[19]. It has been reported that the interaction of RANK with RANKL results in the activation of distinct intracellular pathways: NFATc1, NF-κB, Akt, and MAP kinase pathways. Osteoprotegerin (OPG) acts as a decoy receptor for RANKL and blocks RANKL-mediated osteoclast differentiation and stimulation of osteoclast resorbing activity [20], [21]. Although RANKL is a crucial factor for osteoclastogenesis, RANKL-independent mechanisms have been evidenced with several pro-inflammatory cytokines such as TNF-α, LIGHT and IL-8 [22]–[25].

The aim of the present study was to investigate the role of IL-32 on the differentiation and maturation of human osteoclast precursors. Our findings suggested that although IL-32 induced fusion of osteoclast precursors into multinucleated TRAcP+, VNR+ cells expressing OSCAR and Cathepsin K and induced the activation of NFATc1, NF-κB, ERK1/2 and JNK, it was incapable of modulating Akt activation in a level similar to RANKL. Morphologically multinucleated cells generated in response to IL-32 did not form F-actin on dentine slices and ultimately were unable to resorb bone *in vitro*.

## Results

### IL-32 induces the differentiation of adherent PBMCs into multinucleated TRAcP+, VNR+ cells in the absence of sRANKL

**Figure 1:**
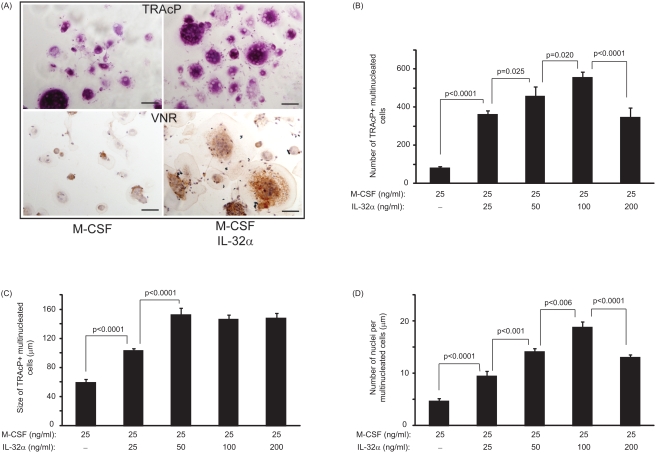
IL-32-treatment induced a dose dependent differentiation of osteoclast precursors. (A) TRAcP and VNR positive staining of newly-formed multinucleated cells differentiated from human PBMCs in the presence of 25 ng/ml of M-CSF±100 ng/ml of IL-32. Black bar represents 50 µm. (B) The number of newly-generated TRAcP positive multinucleated cells is dependent on the concentration of IL-32. (C) Size of newly-generated multinucleated cells is not increased at doses higher than 50 ng/ml of IL-32. (D) The number of nuclei per newly-generated multinucleated cells is significantly increased in a dose-dependent manner with the optimal response at 100 ng/ml of IL-32. P values represent the statistical significances between each group using Mann-Whitney test.

After 14 days in culture, the presence of newly-formed multinucleated cells were detected in response to IL-32. To determine the optimal concentration of IL-32 required to differentiate PBMCs into multinucleated cells *in vitro*, a dose response experiment was performed. IL-32 was capable of inducing the formation of multinucleated TRAcP and VNR positive cells as shown in Figure 1A. The number of TRAcP positive multinucleated cells was increased by a significant 4.5-fold at the lowest concentration studied (25 ng/ml) compared to M-CSF alone (361±12 vs. 79±5; p<0.0001) as seen on Figure 1B. The dose dependent effect of IL-32 was statistically significant and clearly evident as illustrated in Figure 1B. A similar pattern for the number of VNR positive multinucleated cells in response to IL-32 was noted (data not shown).

The size of newly-formed multinucleated TRAcP+ VNR+ cells in response to IL-32 was also significantly greater than in M-CSF-treated culture (Fig. 1C). Interestingly, at doses higher than 50 ng/ml, IL-32 did not markedly influence the size of the newly-formed multinucleated TRAcP+ VNR+ cells. The number of nuclei per multinucleated cells was significantly increased in IL-32-treated cultures compared to M-CSF alone with a 2-fold increase for the lowest concentration of 25 ng/ml (9.44±2.74 vs. 4.66±1.36; p<0.0001). The dose dependent effect of IL-32 was clearly evident as illustrated in Figure 1D. Based on the data obtained in Figures 1B–1D, the optimal dose for any subsequent experiments was chosen to be 100 ng/ml.

### IL-32 is capable of inducing the formation of multinucleated cells exhibiting specific osteoclast markers

**Figure 2:**
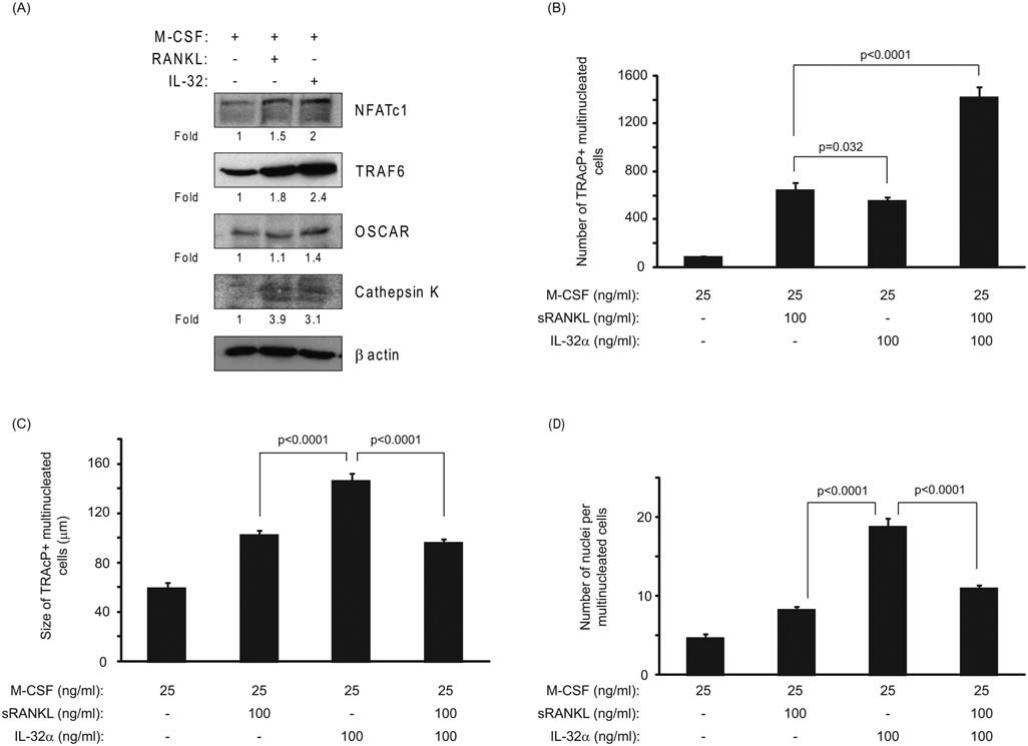
Comparison of the effects of IL-32 and soluble RANKL on the differentiation and maturation of osteoclast precursors. (A) IL-32 was capable of inducing the expression of specific osteoclastic markers as illustrated. PBMCs were exposed to the factors for 72 h prior to Western blot analysis. β-actin was used as an internal control for gel loading. (B) Number of newly formed osteoclasts. Although the number of multinucleated cells formed in response to IL-32 was significantly increased compared to M-CSF-treated cultures, this parameter was significantly decreased when compared with the soluble RANKL-treated cultures. The combined treatment of PBMCs with sRANKL/IL-32 resulted in a highly significant increase in the number of newly formed osteoclasts as compared to either of those factors alone. (C) Size of newly generated osteoclasts. IL-32 increased the size of multinucleated cells compared to sRANKL cultures but was unable to increase the size of the newly formed osteoclasts when combined with sRANKL. (D) Number of nuclei per osteoclast. Compared to sRANKL-treated cultures, multinucleated cells formed in response to IL-32 exhibited more nuclei per cell indicating that the process of cell fusion may have been facilitated. P values represent the statistical significances between each group using Mann-Whitney test.

After 3 days of treatment with M-CSF/IL-32 or M-CSF/RANKL (as a positive control), the expression of specific osteoclast markers such as NFATc1, TRAF6, OSCAR and Cathepsin K, were up-regulated as compared to M-CSF treatment alone (Fig. 2A). The number of newly-generated multinucleated cells formed in the presence of IL-32 was statistically lower than those formed in response to sRANKL (645±37 vs. 555±18; p = 0.032) (Fig. 2B). In contrast, the size and the number of nuclei of the newly-formed multinucleated cells in response to IL-32 were significantly increased by 1.4-fold and 2.3-fold respectively compared to those generated in the presence of sRANKL (146±6.4 µm vs. 102±3.4 µm; p<0.0001 and 18.8±2.5 vs. 8.2±1.3; p<0.0001) (Fig. 2C and 2D).

When a combination of IL-32 and sRANKL (both at 100 ng/ml) were added to the osteoclast precursors, the number of newly-formed osteoclasts was increased by 2.2-fold compared to sRANKL-treated cultures (1417±51 vs. 645±37; p<0.0001) (Fig. 2B). Paradoxically, the size of the newly-formed osteoclasts in response to IL-32 and sRANKL was significantly reduced compared to IL-32-treated cultures (96±3 µm vs. 146±6.4 µm; p<0.0001). However, compared to sRANKL-treated cultures, the size of TRAcP positive multinucleated osteoclasts were not significantly different as those formed in the presence of IL-32 and sRANKL (96±3 µm vs. 102±3.4 µm; p = 0.310) (Fig. 2C). The number of nuclei per newly-formed osteoclast was significantly reduced in PBMC cultures treated with combined sRANKL/IL-32 as compared to IL-32 alone (11±1.1 vs. 18.8±2.5; p<0.0001) (Fig. 2D).

### Signalling pathways involved in response to IL-32 and compared with RANKL

**Figure 3:**
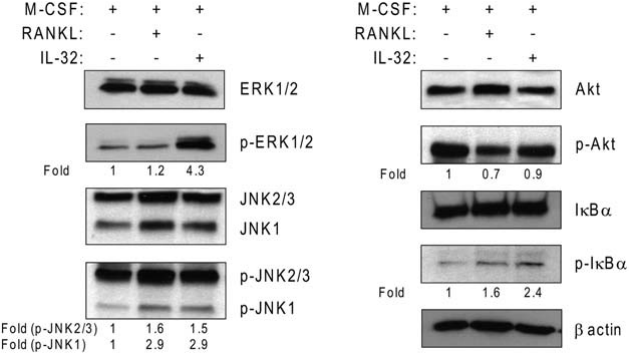
Effects of IL-32 on signalling pathways involved during osteoclastogenesis. Phosphorylation of ERK1/2, JNK, Akt and IkB-α in human PBMCs after 15 minutes exposure to RANKL or IL-32. β-actin was used an internal control of gel loading.

In order to better characterize the multinucleated cells formed in response to IL-32, we investigated, using Western blot analysis, the activation of downstream signalling pathways. After 15 minutes of treatment with M-CSF or RANKL or IL-32, the activation of several intracellular pathways known to be activated by RANKL, were determined. In response to IL-32 or RANKL, PBMCs underwent a rapid activation of JNK and NF-κB as compared to M-CSF-treated cultures (Fig. 3). The phosphorylation of ERK1/2 was markedly increased by 3.6-fold in response to IL-32 compared with RANKL. Although M-CSF alone induced a strong and rapid activation of Akt, the addition of RANKL was incapable of activating Akt in a similar level, indicating a possible down-regulation of Akt activation by RANKL when associated with M-CSF (Fig. 3). Interestingly, Akt activation in IL-32 treatment increased considerably compared to RANKL exposure.

### IL-32 alone cannot induce the maturation of the newly-formed osteoclast into bone –resorbing osteoclasts

**Figure 4:**
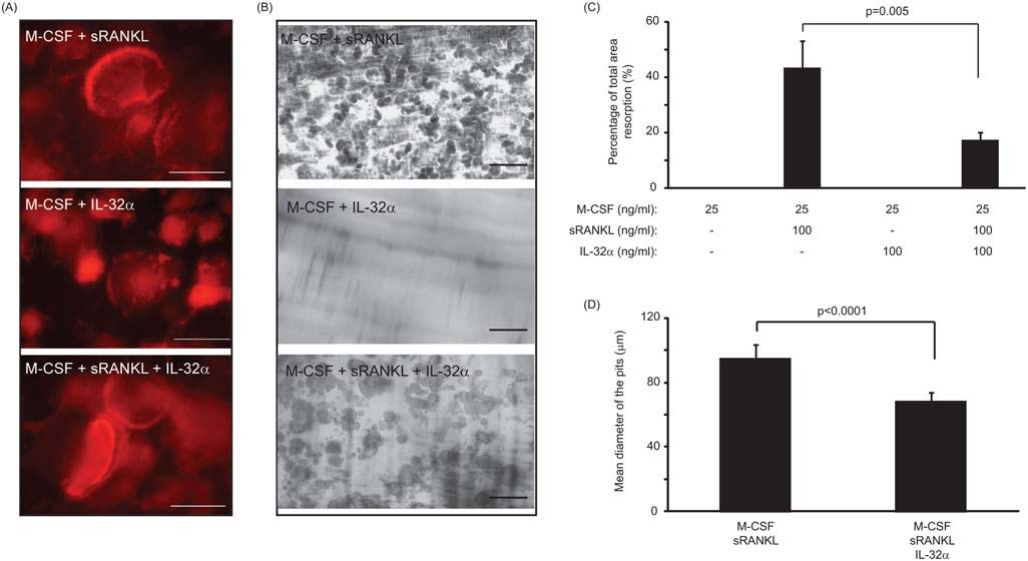
IL-32 was unable to induce the maturation of osteoclasts *in vitro*. (A) F-actin staining of multinucleated cells cultured with M-CSF and sRANKL, M-CSF and IL-32 and M-CSF, sRANKL and IL-32. White bar represents 80 µm. IL-32-treated cultures did not exhibit evidence of F-actin ring formation indicating the lack of anchorage of osteoclasts on dentine surface. (B) Evidence of lacunar resorption on dentine slices cultured with M-CSF and sRANKL, M-CSF and IL-32 and M-CSF, sRANKL and IL-32. Black bar represents 250 µm. Multinucleated cells formed in response to IL-32 were incapable of lacunar resorption. (C) Treatment of PBMCs with M-CSF and IL-32 failed to induce the maturation of newly-formed osteoclasts as evident by the lack of lacunar resorption on dentine slices. However, sRANKL-treatment of PBMCs resulted in a 43.3±9.8% percentage area resorption. Combined treatment of sRANKL and IL-32 resulted in a significant decrease of 2.6 fold in percentage area resorption compared to sRANKL alone. (D) The mean diameter of the lacunar pits formed in response to sRANKL and IL-32 treatment was significantly decreased compared to those generated in response to sRANKL alone. P values represent the statistical significances between each group using Mann-Whitney test.

In order to determine whether IL-32 alone or in combination with sRANKL have a direct effect on the maturation of osteoclasts, the expression of F-actin ring by the newly-formed multinucleated cells and the extent of lacunar resorption was examined on dentine slices of PBMC cultures after 21 days. Multinucleated cells generated in the presence of sRANKL or in combination of sRANKL/IL-32 presented an F-actin ring at their periphery as shown in Figure 4A. However, multinucleated cells generated in response to IL-32 did not similarly express F-actin ring (Fig. 4A). Furthermore, IL-32 alone was shown to have no effect on the maturation of newly-formed osteoclast as indicated in Figure 4B and 4C. However, although the mean percentage surface area resorption in response to sRANKL alone was 43.3±9.8%, the addition of IL-32 in combination to sRANKL did not increase the extent of lacunar resorption and surprisingly it significantly reduced this parameter by 2.6-fold (Fig. 4C).

To ascertain the mechanism by which IL-32 induced the observed decrease in the percentage lacunar resorption in sRANKL-treated cultures, the size of the lacunar pits were determined by image analysis. In cultures treated with IL-32 and sRANKL the mean diameter of the pits was significantly reduced by 1.4-fold compared to sRANKL alone (68.3±1.0 µm vs. 94.7±1.6 µm; p<0.0001) (Fig. 4D).

### Multinucleated cells formed in response to IL-32 are not solely mediated through the RANK/RANKL pathway

**Figure 5:**
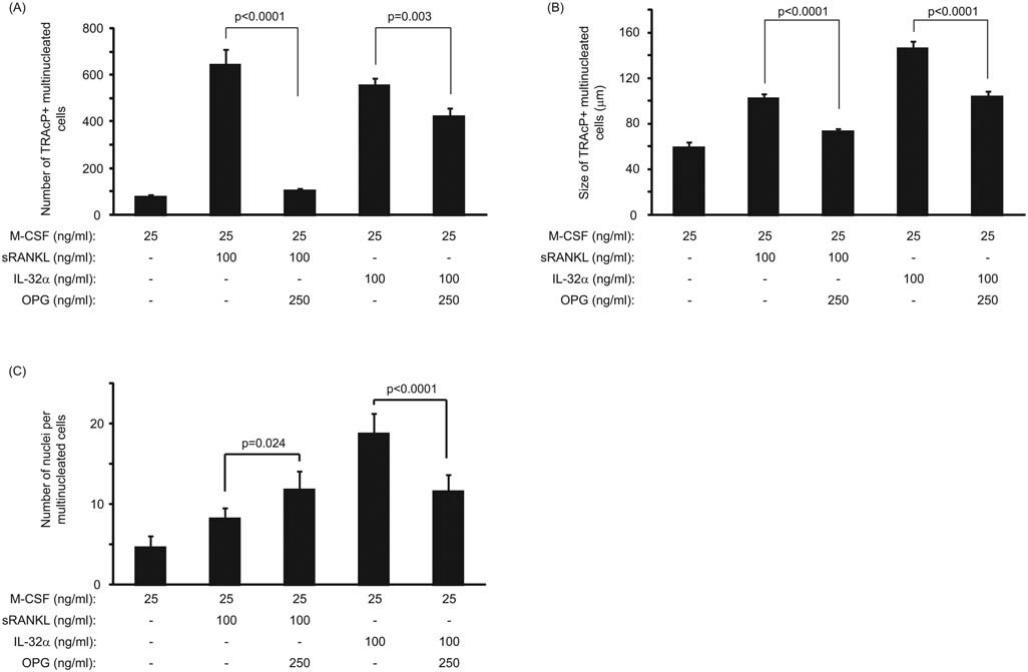
Effect of OPG treatment on sRANKL- and IL-32-mediated osteoclastogenesis. (A) Number of newly-generated osteoclasts in the presence of OPG. OPG significantly decreased the number of osteoclasts generated with sRANKL but OPG inhibition had only a partial effect in PBMC cultures treated with IL-32. (B) Size of the newly-generated osteoclasts. OPG treatment resulted in a decrease in the size of the newly-formed osteoclasts generated with sRANKL and/or IL-32. (C) Number of nuclei per osteoclast. OPG treatment significantly increased the number of nuclei per osteoclast in sRANKL-treated cultures and markedly abrogated this parameter in IL-32-treated cultures. P values represent the statistical significances between each group using Mann-Whitney test.

Figure 5 demonstrates the inhibitory effects of excess OPG on sRANKL-mediated and IL-32-mediated osteoclastogenesis. OPG at 250 ng/ml was capable of significantly inhibiting the number of RANKL-inducing osteoclast formed by 6.2-fold as compared to sRANKL alone (104±5 vs. 645±37; p<0.0001) (Fig. 5A). The size of the osteoclast generated in the presence of sRANKL and OPG was significantly reduced as compared to sRANKL alone (73±2.2 µm vs. 102±3.4 µm; p<0.0001) (Fig. 5B) but the number of nuclei per osteoclast was significantly increased (Fig. 5C). Although the concentration of OPG employed was unable to completely inhibit the number of osteoclasts formation in RANKL-treated cultures, this dose was suitable in completely abolishing the activity of the newly-formed osteoclasts in the resorption assay (data not shown).

OPG treatment resulted in a moderate inhibition (1.3-fold) of the newly-formed multinucleated cells in response to IL-32 as compared to cultures where no exogenous OPG was added (423±22 vs. 555±18; p = 0.003, Fig. 4A). Moreover, OPG was capable of reducing the size and the number of nuclei per newly-formed multinucleated cells generated in response to IL-32 (104±3.9 µm vs. 146±6.4 µm; p<0.0001 and 11.6±2 vs. 18.8±2.5; p<0.0001, Fig. 5B). As the extent of lacunar resorption did not mirror the number of multinucleated cells formed in cultures treated with IL-32 (Fig. 4B), the addition of OPG to these cultures did not affect the surface area resorption by IL-32 as there was no evidence of pit formation in response to this cytokine (data not shown).

### OPG inhibits osteoclast formation and resorption in sRANKL/IL-32 combined treatment

**Figure 6:**
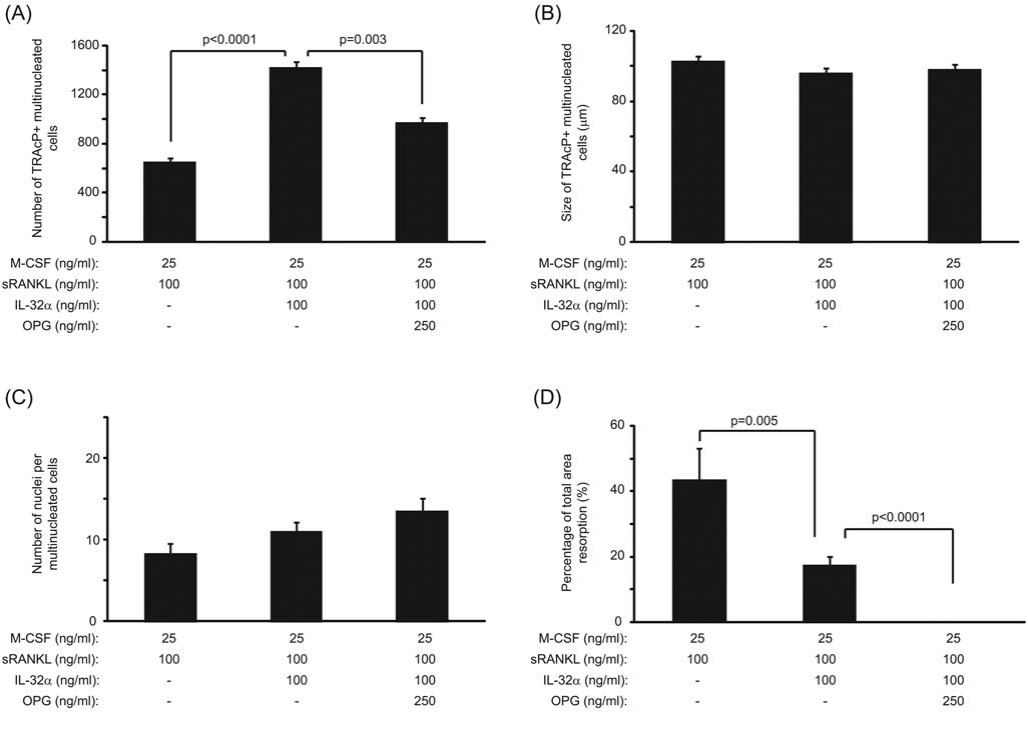
Effects of OPG on IL-32/sRANKL-mediated osteoclastogenesis. (A) The number of multinucleated TRAcP positive cells formed in response to IL-32/sRANKL treatment was significantly reduced in the presence of excess OPG. (B) The size of the newly-formed TRAcP positive multinucleated cells was unaffected by the presence of OPG in IL-32/sRANKL-treated cultures. (C) The number of nuclei noted in TRAcP positive cells formed in response to IL-32/sRANKL treatment was not affected by the OPG treatment. (D) The percentage area lacunar resorption was completely abolished when PBMCs were treated with IL-32/sRANKL and excess OPG. P values represent the statistical significances between each group using Mann-Whitney test.

In cultures treated with IL-32 and sRANKL, the number of multinucleated osteoclasts formed was markedly reduced in response to excess concentration of OPG as compared to untreated cultures (Fig. 6A). The size and the number of nuclei of the newly-formed osteoclasts were, however, unaffected in cultures treated with sRANKL, IL-32 and OPG as compared to conditions in the absence of OPG (Fig. 6B and 6C). OPG treatment of sRANKL/IL-32 PBMC cultures resulted in a complete inhibition of lacunar resorption as shown in Figure 6D.

### Release of soluble mediators in response to IL-32

**Figure 7:**
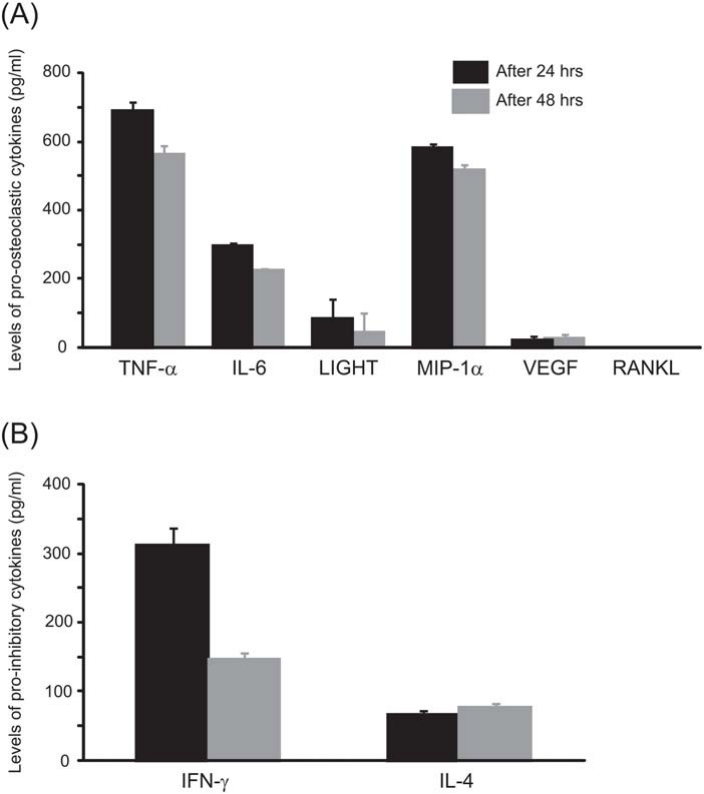
Release of soluble mediators in response to treatment of PBMCs with IL-32 after 24 hrs and 48 hrs. (A) The stimulation of PBMCs with 100 ng/ml of IL-32 induced the release of TNF-α, IL-6, LIGHT, MIP-1α, VEGF. RANKL was undetectable in the supernatant. (B) Levels of IFN-γ and IL-4. The minimum dose detectable was 1.6 pg/ml for TNF-α, 0.70 pg/ml for IL-6, 5.5 pg/ml for LIGHT, 10 pg/ml for MIP-1α, 5 pg/ml for VEGF, 5 pg/ml for RANKL, 8.0 pg/ml for IFN-γ and 10 pg/ml for IL-4. Unstimulated PBMCs were unable to release any of these soluble mediators.

Figure 7 illustrates the level of cytokines known to induce osteoclastogenesis (Fig. 7A) and factors known to inhibit osteoclast differentiation (Fig. 7B), released in the cell culture supernatant after stimulation by 100 ng/ml of IL-32. After 24 hrs, the levels of TNF-α, IL-6, LIGHT, MIP-1α, VEGF, IFN-γ and IL-4 were significantly higher compared to unstimulated cultures, where these cytokines were not detectable in the culture supernatant. After 48 hrs, a similar pattern of cytokine release was evidenced with the detection of high levels of TNF-α, IL-6, LIGHT, MIP-1α, VEGF, IFN-γ and IL-4. Here again in the unstimulated culture none of these cytokines were evidenced. Soluble RANKL was undetectable in the supernatant of IL-32-treated or unstimulated cultures after 24 hrs and 48 hrs.

## Discussion

Osteoclasts are multinucleated cells responsible for bone resorption and are derived from hematopoietic precursor cells that circulate in the blood [26]–[28]. It is currently thought that two critical factors supplied by osteoblasts, M-CSF and RANKL, are essential for the differentiation and maturation of osteoclast precursors [11], [29]. Although M-CSF defective mice (op/op) show an osteopetrotic phenotype, it can spontaneously reverse with age, suggesting that alternative osteoclastic pathways do exist [30]–[32]. Hepatocyte growth factor (HGF), vascular endothelial growth factor (VEGF) and Flt3 ligand have all been shown to support osteoclast formation in the absence of M-CSF [33]–[35]. Furthermore mice deficient in either RANKL or its receptor RANK also show an osteopetrotic phenotype that is caused by the complete lack of osteoclast in their bones [36], [37]. Although no osteoclasts can be identified in the bone of RANKL or RANK deficient mice, this may not be simply because of the complete failure of osteoclastogenesis. Indeed, RANKL is a survival factor for differentiated osteoclasts [38] and it is plausible that impaired osteoclast differentiation superimposed on a shortened lifespan might also explain the observed phenotype in RANKL or RANK deficient mice [39]. As such, alternative RANKL-independent pathways (e.g. LIGHT, TGFβ and TNF-α) have been reported to support osteoclastogenesis in the presence of excess osteoprotegerin, an inhibitor of RANKL-RANK interactions [23], [24], [40]. The emergence of the osteoimmunology field has demonstrated that activated T cells directly modulate osteoclastogenesis and bone resorption [41], [42], and that T cell products, such as IL-17, TWEAK, GM-CSF and IFN-γ, can regulate osteoclast formation [43]–[45]. The present study sought to determine the role of IL-32, a newly described cytokine presenting characteristic of pro-inflammatory cytokine and involved in a variety of inflammatory disorders [1], [6]–[10] in osteoclast differentiation and activation. We have demonstrated that multinucleated cells formed in response to IL-32 expressed several specific markers of osteoclast such as activation of the NF-κB and MAP kinase pathways, expression of TRAcP and VNR, up-regulation of NFATc1, OSCAR and Cathepsin K which were also observed in RANKL-treated cultures. However, IL-32-treated multinucleated cells were unable to induce bone resorption *in vitro*. One explanation for the lack of bone resorption could be attributed to the failure of the multinucleated cells generated in response to IL-32 to express F-actin ring which ultimately is required for anchorage of the cells prior to bone resorption.

An important step in the process of osteoclast differentiation is the induction of NFATc1 above a critical threshold [39]. This is consistent with the findings that overexpression of NFATc1 is sufficient to induce osteoclast differentiation [46]. In the present study, IL-32 was capable of up-regulating the expression of NFATc1 compared to M-CSF-treated cultures or even M-CSF/RANKL-treated cultures. Moreover, in response to IL-32, these cells expressed high levels of OSCAR and Cathepsin K, two markers that are specific for osteoclasts [47], [48]. According to the different markers expressed by the cells in response to IL-32, it is reasonable to conclude that these multinucleated cells are likely to be “immature osteoclasts”. Recently, Kim et al. [39] have reported that TNF-α is capable of inducing multinucleation of osteoclast precursors and expression of osteoclast phenotypic markers (TRAcP, F-actin) in RANK-deficient cells, but was unable to induce evidence of lacunar bone resorption. In the present study, although PBMCs released pro-osteoclastic mediators (TNF-α, IL-6, LIGHT, MIP-1α and VEGF), the lack of lacunar resorption in IL-32-treated cultures could be explained by the fact that IL-32 stimulated the release of known osteoclastic inhibitors, i.e. IL-4 and IFN-γ. Our results herein indeed indicated that this is the case as we demonstrated a significant increase in the release of IL-4 and IFN-γ from IL-32-stimulated PBMCs as compared to unstimulated cells.

Tumour necrosis factor receptor-associated factor 6 (TRAF6) has been reported to be important for osteoclast activation, i.e. lacunar bone resorption [49]. The complex role of IFN-γ in osteoclastogenesis has been previously addressed by Takayanagi *et al.* [50]. They have shown that IFN-γ induces rapid degradation of TRAF6, which results in strong inhibition of the RANKL-induced activation. We therefore speculated that the inhibitory effects of IL-32 alone or in combination with soluble RANKL could partly be attributed to TRAF-6 degradation. However, surprisingly, we found that TRAF6 is not degraded but it is overexpressed in response to IL-32 treatment compared to RANKL. Recently, Gao et al. have shown that IFN-γ exhibits a “direct” anti-resorptive effect by blunting osteoclast differentiation. However, IFN-γ can act “indirectly” as a pro-resorptive factor by stimulating T-cells to express RANKL and TNF-α [51]. In the present study, we used PBMCs as a source of osteoclast precursors and although cells were washed thoroughly to eliminate non-adherent cells (mainly B- and T-cells), it is plausible that some T-cells could have been present in the culture and contributed to osteoclastogenesis. This hypothesis is also reinforced by the evidence that the addition of excess OPG to the IL-32-treated cultures led to a marked decrease in the number and size of newly-formed multinucleated cells. Although we were unable to detect any soluble RANKL in the supernatant of IL-32-treated cultures, it is conceivable to suggest that effects of IL-32 could have been partially mediated through a RANKL-dependent mechanism. We have previously observed that IL-32 increase the expression of membrane-bound RANKL in T-cell cultures (unpublished data) and it is likely that a few numbers of RANKL-expressing T-cells may have been present in the PBMC cultures. As IL-32 is known to be produced by PBMCs in response to IFN-γ [52], the effects of IFN-γ in combination with IL-32 on T-cells could have contributed to the inhibitory effects of OPG observed herein.

**Figure 8:**
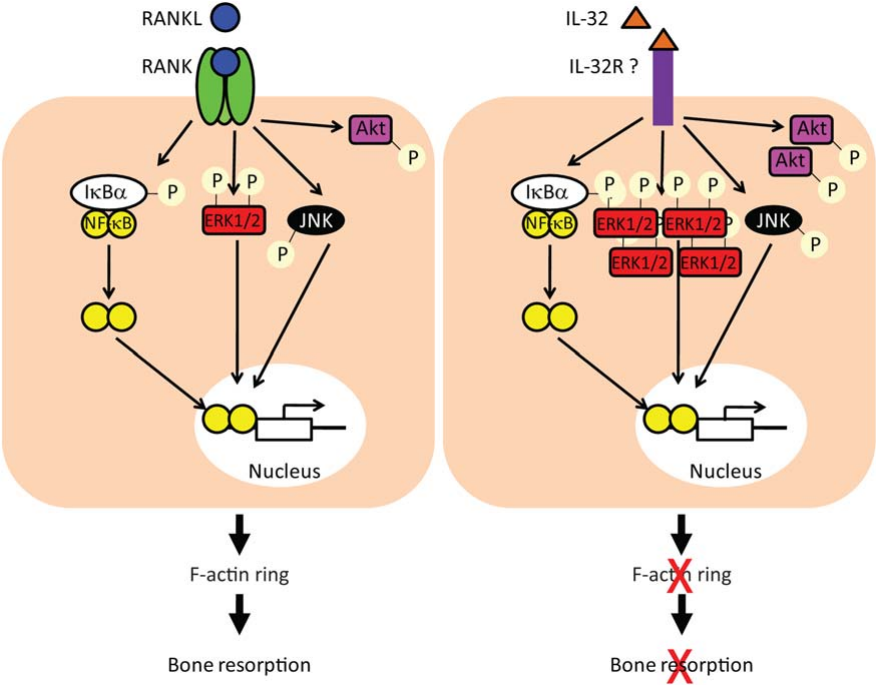
Schematic representation of downstream pathways activated by RANKL or IL-32 treatment. The discrepancy observed between IL-32 and RANKL signalling pathways (i.e. increased ERK1/2 and Akt activation by IL-32) may lead to the activation of different downstream targets which in turn could contribute to the inability of cells to express F-actin ring and resorb in response to IL-32.

The downstream signalling of RANK/RANKL interactions has been extensively studied in the last decade. It has been shown that the binding of RANKL to its receptor activated NF-κB, MAP kinase and Akt pathways. However, downstream pathways involved in response to IL-32 in osteoclasts are not fully elucidated. In our study, we found that PBMCs treatments with M-CSF/IL-32 or M-CSF/RANKL dramatically increased the activation of NF-κB and JNK pathways compared to M-CSF-treated cultures. However, the activation of the Akt pathways appeared more complicated. M-CSF or M-CSF/IL-32 treatments were capable of strongly activating Akt pathways compared to M-CSF/RANKL. These results appeared controversial with the consensus that RANKL activates Akt [11], [53]. However, most of the studies showing Akt activation in response to RANKL have been done in *in vitro* models of bone marrow co-cultures or using murine cell line, RAW264.7 cells. In these studies, treatment of serum-starved osteoclast precursors with soluble RANKL resulted in a significant activation of the Akt pathway as compared to cultures in the absence of RANKL. This is in conflict with the present findings whereby after exposure of M-CSF–treated PBMCs to sRANKL, Akt activation was down-regulated compared with M-CSF alone. Interestingly, M-CSF/IL-32 treatment of PBMCs resulted in a similar level of Akt activation compared to M-CSF treatment. It has also been extensively shown that M-CSF, via its receptor c-fms, is a strong activator of PI-3 Kinase which in turn activates Akt [54], [55]. In the present study, we demonstrated that M-CSF/IL-32 treatment exhibited levels of Akt activation similar to M-CSF treatment and it is questionable whether Akt activation in response to IL-32 or RANKL results in the same downstream effectors (Fig. 8).

We have also evidence of a marked increase in the phosphorylation of ERK1/2 in M-CSF/IL-32 compared to M-CSF/RANKL treated cultures. It is well known that activated ERK1/2 translocates to the nucleus and activates its target to promote the expression of specific genes (reviewed in [56]). It is plausible to suggest that the downstream targets of activated ERK1/2 in M-CSF/IL-32 are different to those known for M-CSF/RANKL (Fig. 8). This is also reinforced by the morphological differences (e.g. differences in cell size and number of nuclei, lack of F-actin ring) observed in the multinucleated cells generated in presence of IL-32 or RANKL.

Our current findings suggest that IL-32 was capable of inducing osteoclast differentiation in a manner partially independent of the RANK/RANKL pathway. However, although IL-32 could increase the release of pro-inflammatory mediators known to positively influence osteoclastogenesis, it was unable to induce the activation of these newly-formed multinucleated cells into bone-resorbing osteoclasts and had a direct inhibitory effect on osteoclast activation *in vitro*. It is worth noting that IL-32 has direct effects on other cell types such as epithelial cells, T-cells, natural killer cells and monocytes. Although the present study only addressed its direct role on osteoclast precursors, IL-32 could also indirectly modulate osteoclastogenesis *in vivo*. The complex role of IL-32 in the patho-physiology of bone disorder therefore requires further clarification prior to the development of any potential therapeutic approach.

## Materials and Methods

Recombinant IL-32α, M-CSF and OPG were purchased from R&D systems Europe (Abingdon, UK) and soluble RANKL was purchased from PeproTech (London, UK). Recombinant cytokines were aliquoted and stored at −80°C on day of purchase. All chemicals for immuno-histochemical staining were purchased from Sigma-Aldrich (Poole, UK).

### Preparation and culture of human peripheral blood mononuclear cells (PBMCs)

The study was approved by the Oxfordshire Research Ethics Committee (CO1-071). PBMCs were isolated from 6 normal healthy volunteers as previously described [57]. Briefly, donors were free of prescribed and over the counter prescription. Antecubital venous blood was supplied by National Blood Service (UK). Blood was diluted 1∶1 in α-minimal essential medium (MEM) (Invitrogen, Paisley, UK), layered over Histopaque (Sigma-Aldrich Chemicals, Poole, UK), and centrifuged (693× g) for 20 min. The interface layer was resuspended in MEM then centrifuged (600× g) for a further 10 min after which the resultant cells were resuspended in media supplemented with 10% heat inactivated fetal calf serum (FCS, Invitrogen, Paisley, UK) and counted in a haemocytometer following lysis of red blood cells by a 5% (v/v) acetic acid solution.

### Assessment of osteoclast formation

To assess the extent of osteoclast formation and activation, isolated human PBMCs from whole blood were cultured on glass coverslips and dentine slices as described previously [57]. Briefly, 5×10^5^ PBMCs were added to 4 mm diameter dentine slices and 6 mm diameter glass coverslips in MEM containing 100 UI/ml penicillin, 100 µg/ml streptomycin and 10% FCS. After 2 h incubation, cultures were vigorously rinsed in medium to remove non-adherent cells, and then maintained in 1 ml MEM/FCS with 25 ng/ml recombinant human M-CSF with or without 100 ng/ml recombinant human sRANKL for up to 21 days. To assess whether IL-32 could influence osteoclastogenesis, PBMCs were cultured in the following conditions:

- 25 ng/ml of M-CSF (negative control)
- M-CSF (25 ng/ml) and sRANKL (100 ng/ml) as a positive control (sRANKL added on day7)
- M-CSF (25 ng/ml) and various concentration of IL-32 (25 ng/ml, 50 ng/ml, 100 ng/ml and 200 ng/ml) (IL-32 added on day7)
- M-CSF (25 ng/ml), 100 ng/ml sRANKL and 100 ng/ml IL-32 (the latter two added on day7)
- M-CSF (25 ng/ml), sRANKL (100 ng/ml) and 250 ng/ml OPG, a dose known to completely block the sRANKL-mediated bone resorption [58] (both sRANKL and OPG added at day7).
- M-CSF (25 ng/ml), 100 ng/ml IL32-α and 250 ng/ml OPG to determine the possible involvement of the sRANKL pathway (IL-32 and OPG added on day7).
- M-CSF (25 ng/ml), 100 ng/ml IL-32, 100 ng/ml sRANKL and 250 ng/ml OPG (IL-32, sRANKL and OPG added at day7).

All factors were replenished every 2–3 days and the cultures were maintained for up to 21 days.

### Characterization of newly-formed osteoclast on glass coverslips and dentine slices

#### Tartrate resistant acid phosphatase (TRAcP)

After 14 days in culture, the expression of TRAcP (one of the known osteoclastic markers [59]) on the adherent cells on the coverslips were examined histochemically, as described previously [59]. Briefly, coverslips were removed from the culture wells and rinsed promptly in PBS buffer, fixed with formalin (10% in PBS buffer) for 10 minutes and rinsed in distilled water. TRAcP was histochemically revealed by a simultaneous coupling reaction using Naphtol AS-BI-phosphate as substrate and Fast violet B as the diazonium salt. The coverslips were then incubated for 90 minutes at 37°C in the dark, rinsed three times in distilled water and the residual activity was inhibited by 4% NaF for 30 minutes. Coverslips were then rinsed in distilled water, counterstained with DAPI for 20 minutes and allowed to dry before mounting using an aqueous medium. TRAcP positive cells, with more than three nuclei, were considered as osteoclasts. The number of newly generated osteoclasts and the number of nuclei per osteoclast (after counterstaining with DAPI) were assessed using light microscopic examination. The size of the osteoclasts was determined by image analysis using the ImageJ Freeware (NIH, Bethesda, MD).

#### Vitronectin receptor (VNR)

Adherent cells on coverslips were fixed with 4% paraformaldehyde after 14 days of culture and stained immuno-histochemically by an indirect immunoperoxidase technique. This technique utilized the monoclonal antibody 23C6 (Serotec, Oxford, UK), which is directed against the VNR, CD51, an osteoclast-associated antigen [60].

#### F-actin

Adherent cells on dentine slices were fixed in 4% paraformaldehyde after 21 days of culture, washed in PBS and stained histo-chemically with phalloidin-TRITC (500 ng/ml) for 45 minutes. Dentines slices were washed thoroughly in PBS prior to mounting using 70∶30 (v/v) water∶glycerol and examined with an Olympus BX40.

#### Lacunar resorption

After 21 days in culture, the dentine slices were removed from the culture wells, placed in NH_4_OH (1N) for 30 minutes and sonicated for 5 minutes to remove any adherent cells. After rinsing in distilled water, the dentine slices were stained with 0.5% (v/v) toluidine blue (pH 5.0) prior to examination by light microscopy. The surface of each dentine slice was examined for the presence of lacunar resorption and the extent of eroded surface on dentine slices was determined using image analysis on each dentine and data was expressed as the percentage of surface area resorbed.

### Western blot analysis

PBMCs were cultured on 25 cm^2^ flasks with M-CSF (25 ng/ml) alone or M-CSF and soluble RANKL (100 ng/ml) or M-CSF and IL-32 (100 ng/ml) for the indicated period of time. Cells were washed in cold PBS and homogenised in lysis buffer containing 50 mM Tris-Hcl pH 7, 100 mM NaCl, 50 mM NaF, 3 mM Na_3_VO_4_, protease inhibitor cocktail (Sigma-Aldrich) and 1% Nonidet P-40. Samples were spun at 13,000 rpm for 30 min at 4°C, the supernatant was collected and protein concentration was determined using a BCA protein assay kit (Pierce Biotechnology, Rockford, IL). Samples (20–50 µg per lane) were run on a 10% acrylamide and blotted onto a PVDF membrane. The membranes were washed in Tris buffered saline (TBS) and blocked with 5% bovine serum albumin. Samples were incubated overnight with one of the following specific antibodies for IκBα, Phospho-IκBα, ERK1/2, Phospho-ERK1/2, JNK, Phospho-JNK, Akt, Phospho-Akt, TRAF6 (Cell Signalling), Cathepsin K, NFATc1, OSCAR (Santa Cruz Biotechnology) and β-actin (Sigma-Aldrich). Subsequently the membranes were washed in TBS and incubated with the appropriate secondary antibodies coupled to HRP. Immunoreactive bands were visualised using an ECL kit (Amersham, UK). The degree to which the different markers were induced was determined by normalizing the specific signal to that of actin using ImageJ Freeware (NIH).

### Cytokine release in response to IL-32

In order to determine the extent of different cytokine release involved in the osteoclastogenic process, the condition media of 2×10^6^ PBMCs exposed to 100 ng/ml of IL-32, a concentration that was shown to influence osteoclastogenesis (see above), was collected after eight and nine days in culture (i.e. after one and two days of presence with IL-32). These time points were chosen in order to determine whether IL-32 is capable of inducing the release of other known osteoclastogenic markers (listed below) directly from PBMCs already stimulated by M-CSF. The conditioned media was thereafter collected, centrifuged at 800× g for 30 min, aliquoted and frozen at −80°C prior to evaluation of the levels of human IFN-γ, IL-4, IL-6, LIGHT, MIP-1α also known as CCl-3, TNF-α and VEGF (Human Quantikine Kits, R&D Systems Europe, Abingdon, UK) according to the manufacturer protocol. According to the manufacturer, the minimum dose detectable (MDD) of these cytokines is 8.0 pg/ml for IFN-γ, 10 pg/ml for IL-4, 0.70 pg/ml for IL-6, 5.5 pg/ml for LIGHT, 10 pg/ml for MIP-1α, 1.6 pg/ml for TNF-α and 5 pg/ml for VEGF. Levels of soluble RANKL were detected using in-house kit by coating a 96-well plate with recombinant human OPG (R&D Systems Europe) and using a goat anti-human RANKL (R&D Systems Europe) as a detection antibody. The specificity of the kit was validated using recombinant soluble RANKL from PeproTech (range from 0 to 1280 pg/ml). The minimum dose detectable of RANKL is 5 pg/ml.

### Statistical analysis

All experiments were repeated at least 6 times and the results were expressed as mean±standard error of the mean. Non-parametric Mann-Whitney test was used to compare the differences between the groups using the SPSS statistical software release 14.0. (SPSS inc. Chicago, IL). Differences at p<0.05 were considered significant.
